# An Unusual Case of Pancreatic Metastasis from Squamous Cell Carcinoma of the Lung Diagnosed by EUS-Guided Fine Needle Biopsy

**DOI:** 10.1155/2017/3212056

**Published:** 2017-05-17

**Authors:** Takuya Ishikawa, Yoshiki Hirooka, Carolin J. Teman, Hidemi Goto, Paul J. Belletrutti

**Affiliations:** ^1^Division of Gastroenterology and Hepatology, Department of Medicine, University of Calgary, Calgary, AB, Canada; ^2^Department of Gastroenterology, Nagoya University Graduate School of Medicine, Nagoya, Japan; ^3^Department of Endoscopy, Nagoya University Hospital, Nagoya, Japan; ^4^Division of Anatomic Pathology and Cytopathology, Department of Pathology and Laboratory Medicine, University of Calgary, Calgary, AB, Canada

## Abstract

We report a case of a 70-year-old man who presented with abdominal pain and weight loss, with initial imaging showing simultaneous mass lesions in the pancreas and lungs along with extensive lymphadenopathy in the thorax up to the left supraclavicular region. Core biopsies of the left supraclavicular lymph node showed squamous cell carcinoma, which required differentiation between secondary and primary pancreatic neoplasms. Endoscopic ultrasound-guided sampling using a novel fine needle biopsy system was key to making a definite histological diagnosis and determining the best treatment plan.

## 1. Introduction

Metastatic tumors in the pancreas are uncommon, and most cases are difficult to distinguish from a primary pancreatic cancer. Accurate identification of isolated pancreatic metastases is critical in determining the best surgical and/or medical management [1]. Although lung cancer can metastasize to the pancreas, the frequency ranges according to the histological subtype, and the incidence of pancreas involvement with squamous cell carcinoma is reported as 1.1% of all pancreatic metastases [2]. Here we report a case of histologically certified metastatic squamous cell carcinoma involving the pancreas from a primary lung cancer, definitively diagnosed with endoscopic ultrasound-guided fine needle biopsy (EUS-FNB).

## 2. Case Report

**Figure 1:**
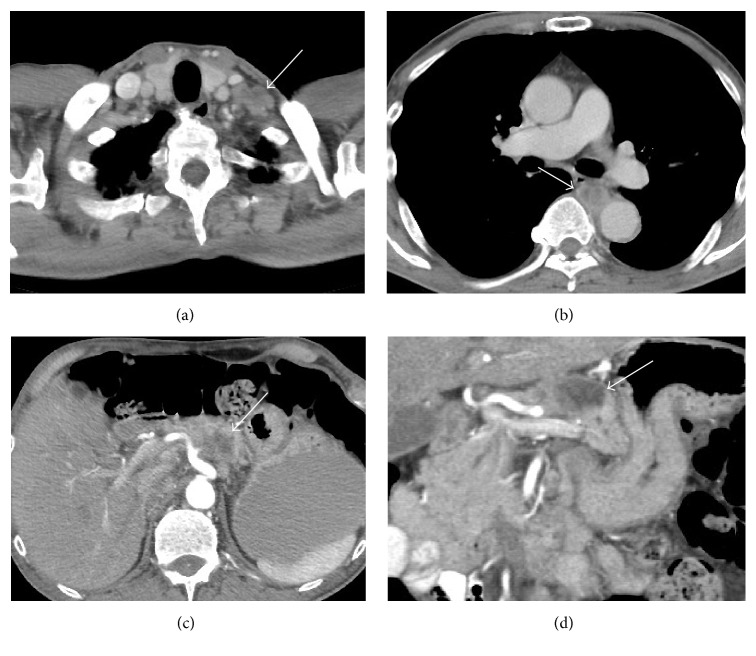
Computed tomography. (a) A conglomerate of lymph nodes in the left supraclavicular fossa measuring 3 cm (arrow). (b) A 2 cm left mid lung mass posterior to the left main stem bronchus (arrow). (c and d) A 3.8 cm hypodense mass in the pancreatic body with associated ill-defined soft tissue inseparable from the distal celiac axis and its branches (arrow).

**Figure 2:**
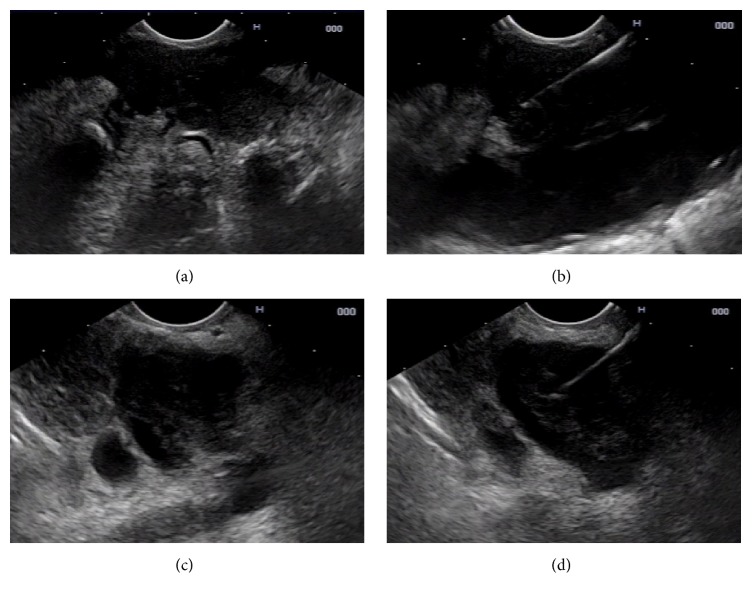
(a) Endoscopic ultrasound (EUS) demonstrating a 3 cm hypoechoic mass in the left lung. (b) EUS-guided fine needle biopsy (FNB) of the left lung nodule with a 25-gauge needle. (c) EUS demonstrating a 3.8 cm hypoechoic mass in the pancreatic body abutting the splenic artery. It shows the same internal echotexture as the lesion in the mediastinum. (d) EUS-FNB of the pancreatic mass with a 25-gauge needle.

**Figure 3:**
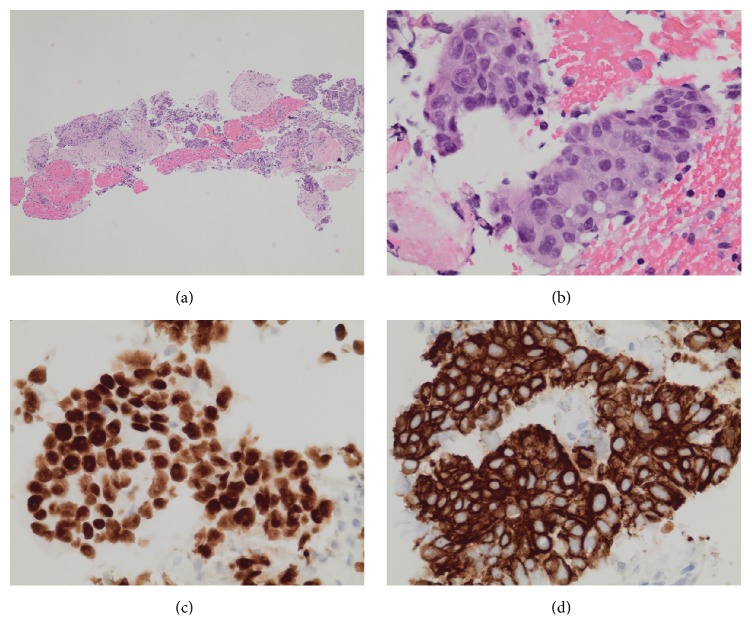
(a) Hematoxylin and eosin staining of a specimen obtained from lung mass with EUS-guided fine needle biopsy. (b) Small core biopsy fragments show invasive carcinoma with clusters and cords of cells that show squamous morphology. (c and d) Immunostains showed that the neoplastic cells express p63 (c) and CK 5/6 (d).

**Figure 4:**
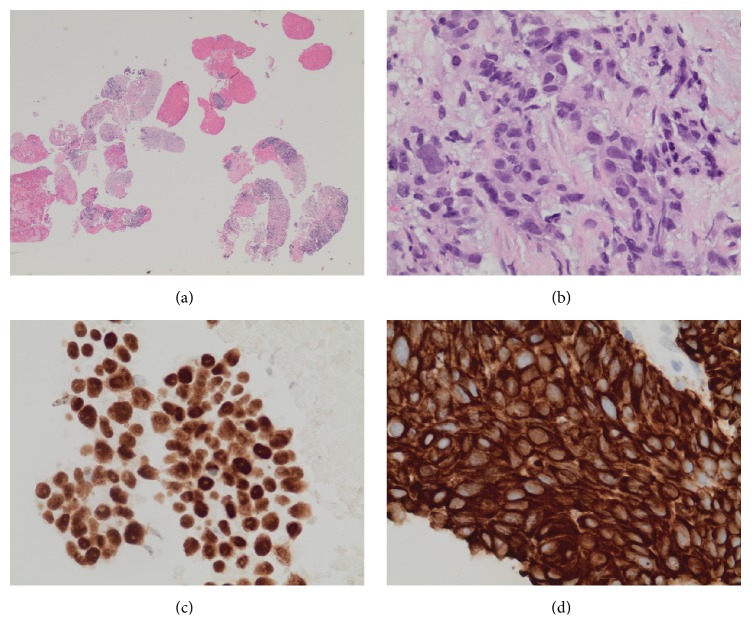
(a) Hematoxylin and eosin staining of a specimen obtained from pancreatic mass with EUS-guided fine needle biopsy. (b) Biopsy fragments show invasive carcinoma morphologically similar to the carcinoma identified in the supraclavicular lymph node and the mediastinal mass. (c and d) Immunostains show that the neoplastic cells express p63 (c) and CK 5/6 (d).

A 70-year-old man presented with a three-month history of progressive abdominal pain and weight loss. His past medical history was unremarkable including no prior history of malignancy. On examination by his primary physician, he was alert and without jaundice or scleral icterus. He had mild epigastric tenderness on abdominal examination, and there were palpable lymph nodes in the left supraclavicular fossa. The remainder of his examination was unremarkable. Laboratory test results were all within normal limits including common blood cell counts, liver chemistries, and serum lipase. Transabdominal ultrasound showed a large distal pancreatic mass. CT scanning revealed a 3.8 cm hypodense mass in the pancreatic body with lymphadenopathy in the left supraclavicular region. It also showed a 3 cm lung mass posterior to the left main stem bronchus (Figure 1). Percutaneous biopsy of one of the left supraclavicular lymph nodes revealed squamous cell carcinoma. The patient was referred to our institution for a tissue diagnosis via endoscopic ultrasound (EUS) of the mediastinal mass and to determine the origin of the pancreas mass. Endoscopically there was no abnormality in the esophagus or the laryngopharynx. EUS using a linear-array echoendoscope (PENTAX EG-3870UTK) revealed two well-defined hypoechoic lesions with similar echotexture firstly in the mediastinum posterior to the left main stem bronchus and secondly in the pancreatic body. EUS-FNB of these two lesions was performed (Figure 2) with a 25-gauge needle using a novel fine needle biopsy system (Beacon SharkCore, Medtronic Corp., Boston, USA). Histopathological examination of both of the specimens revealed a carcinoma morphologically similar to the supraclavicular lymph node biopsy. Immunostains performed on both specimens showed positivity for CK5/6 and p63 (Figures 3 and 4). The patient was thus diagnosed with a metastatic squamous cell carcinoma involving the pancreas from a primary lung cancer. Palliative chemotherapy was planned for the patient.

## 3. Discussion

Clinically apparent pancreatic metastases, while infrequent, are not rare, accounting for up to 3% of solid pancreatic lesions [3]. Although lung cancer is the second most common primary malignancy that metastasizes to the pancreas (next to renal cell carcinoma), the frequency ranges according to the histological subtype [4], and squamous cell carcinoma is extremely rare. The most frequent type is small cell carcinoma with a pancreatic metastasis incidence of 10%, followed by adenocarcinoma (2.4%), large cell carcinoma (1.9%), and finally squamous cell carcinoma with an incidence of only 1.1% [2].

These metastatic lesions are usually asymptomatic or the symptoms are nonspecific. In many cases, the metastatic lesions are discovered incidentally and are mistaken for primary pancreatic tumors. There are no radiological findings that are pathognomonic of pancreatic metastases. On EUS evaluation, it is reported that metastatic lesions are more likely to have well-defined borders compared with primary pancreatic cancer, but, otherwise, EUS features cannot distinguish between the two groups [5]. Therefore, pathological confirmation of pancreatic tumors is the best method for the diagnosis of pancreatic metastases.

The usefulness of EUS-guided tissue sampling in the diagnosis of pancreatic metastases has previously been reported [1, 5–7]. However, some of the metastatic tumors to the pancreas (e.g., esophageal and non-SCLC metastases) cannot be definitively confirmed by cytomorphology alone [5], and obtaining optimal histological samples is highly desirable to improve diagnostic accuracy and certainty. Tissue specimens for histological examination can provide the opportunity to immunostain the tissue, further increasing differential diagnostic capabilities for suspected metastatic lesions. Several new “core” FNA needles have been developed, and one such needle, SharkCore, is attracting attention now. Kandel et al. [8] demonstrated that histological cores were obtained from 95% of the FNB samples compared with 59% of the FNA samples in a total of 156 patients, and the histology yield was significantly higher using the SharkCore needle compared with the EUS-FNA needle (*P* = 0.01). Additionally, a large North American multicenter study of the SharkCore needle was recently reported [9]. In this study, a total of 250 lesions were biopsied in 226 patients, and the overall pathologic diagnostic rate was 88% with a median number of 2 passes. In the present case, the core needle made it possible to obtain adequate histological specimens enabling multiple immunostains to be performed leading to the correct diagnosis and allowing appropriate clinical management to be started without the need for additional time-consuming diagnostic procedures.

In conclusion, although pancreatic metastases from squamous cell carcinoma of the lung are unusual, differentiation from primary pancreatic neoplasms is important. EUS-FNB can allow for a definite histological diagnosis and determination of the most appropriate treatment plan.
